# Peregrine falcons shift mean and variance in provisioning in response to increasing brood demand

**DOI:** 10.1093/beheco/arad103

**Published:** 2023-12-22

**Authors:** Rebekah A McKinnon, Kevin Hawkshaw, Erik Hedlin, Shinichi Nakagawa, Kimberley J Mathot

**Affiliations:** Department of Biological Sciences, University of Alberta, CW 405, Biological Sciences Building, Edmonton, AB T6G 2E9, Canada; Nunavut Wildlife Cooperative Research Unit, Universiy of Alberta, CW 405, Biological Sciences Building, Edmonton, AB T6G 2E9,Canada; Nunavut Wildlife Cooperative Research Unit, Universiy of Alberta, CW 405, Biological Sciences Building, Edmonton, AB T6G 2E9,Canada; Department of Renewable Resources, University of Alberta, GSB 751, Edmonton, AB T6G 0N4, Canada; Department of Renewable Resources, University of Alberta, GSB 751, Edmonton, AB T6G 0N4, Canada; Evolution & Ecology Research Centre and School of Biological, Earth and Environmental Sciences, Biological Sciences North, University of New South Wales, Sydney, NSW 2052, Australia; Department of Biological Sciences, University of Alberta, CW 405, Biological Sciences Building, Edmonton, AB T6G 2E9, Canada; Canada Research Chair in Integrative Ecology, Department of Biological Sciences, CW 405, Biological Sciences Building, University of Alberta, Edmonton, AB T6G 2E9, Canada

**Keywords:** animal behavior, provisioning, raptor, risk sensitivity, variance sensitivity

## Abstract

The hierarchical model of provisioning posits that parents employ a strategic, sequential use of three provisioning tactics as offspring demand increases (e.g., due to increasing brood size and age). Namely, increasing delivery rate (reducing intervals between provisioning visits), expanding provisioned diet breadth, and adopting variance-sensitive provisioning. We evaluated this model in an Arctic breeding population of Peregrine falcons (*Falco peregrinus tundrius*) by analyzing changes in inter-visit-intervals (IVIs) and residual variance in IVIs across 7 study years. Data were collected using motion-sensitive nest camera images and analyzed using Bayesian mixed effect models. We found strong support for a decrease in IVIs (i.e., increase in delivery rates) between provisioning visits and an increase in residual variance in IVIs with increasing nestling age, consistent with the notion that peregrines shift to variance-prone provisioning strategies with increasing nestling demand. However, support for predictions made based on the hierarchical model of tactics for coping with increased brood demand was equivocal as we did not find evidence in support of expected covariances between random effects (i.e., between IVI to an average sized brood (intercept), change in IVI with brood demand (slope) or variance in IVI). Overall, our study provides important biological insights into how parents cope with increased brood demand.

## Teaser

Arctic peregrine falcons adjust their provisioning behavior in response to offspring demand. Parents increase their provisioning rate (i.e., decrease the interval between successive provisioning visits) with increased number of nestlings and with increasing nestling age. However, parents also showed marked increases in the variance in the timing of provisioning visits with increasing nestling age. This finding is consistent with peregrines adopting variance-sensitive provisioning behavior to cope with increased nestling demand.

## INTRODUCTION

Life-history theory predicts that parents should adjust the level of care they provide to offspring in response to changes in brood demand (Trivers 1972; Stearns 1976; Wright and Cuthill 1990; Mathot et al. 2012). For example, parents are expected to increase prey delivery rates as brood demand increases (Bryant 1988; Brodin et al. 2003; Budden and Beissinger 2009). This can come about by parents devoting additional energy to increase prey delivery, such as by flying faster, or limiting self-care behaviors (Simmons et al. 1986; Cairns 1987; Moreno 1987). Additionally, parents may broaden the range of delivered prey by shifting the type of prey delivered away from exclusively preferred prey types or reducing selectivity for larger prey items (Wright et al. 1998; Schrimpf et al. 2012). In doing so, parents may decrease overall nutritional quality of delivered prey in favor of increasing overall energy delivered per unit time (Chiu et al. 2009; Wiebe and Slagsvold 2014).

**Figure 1:**
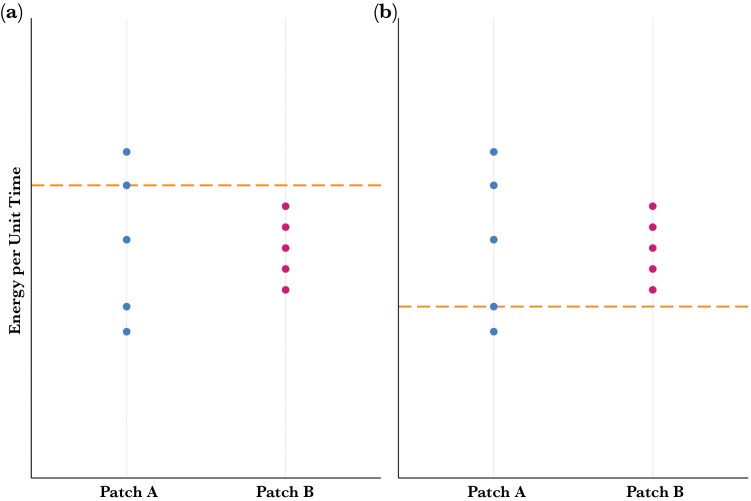
An illustration representing two hypothetical foraging decisions, that is, two patches (A and B) which have the same mean reward but different variance around the mean. (a) Variance-prone: when a forager cannot meet their energetic demands at the less variable patch, they should forage at Patch A. However, (b) Variance-averse: when it is possible for a forager to consistently meet their energetic demands, represented by the dotted line, by foraging at a less variable patch, they should forage at Patch B.

A less commonly appreciated mechanism by which parents can cope with increased brood demand is to exhibit a shift in their preference or aversion for variable foraging options, a behavioral response referred to as variance-sensitivity. Preference for, or aversion to, variable foraging options is influenced by the probability that offspring will experience energetic deficit (Ydenberg et al. 2007; Mathot et al. 2017). When offspring are faced with an average expected intake that is lower than their energy requirements, more variable provisioning options provide a higher probability of starvation avoidance (i.e., survival) compared to less variable options (Figure 1a). Conversely, when there is a low probability of offspring experiencing energetic shortfall and, therefore, a high probability of survival, higher variance comes at a net cost, that is, an increased likelihood of starvation (Figure 1b). When offspring fitness is influenced asymmetrically by deviations above and below the mean delivery rate (Figure 1a and b) (Stephens et al. 2008), parents can use variance as a cue when making provisioning decisions to meet increases in brood demand by strategically shifting their use of provisioning options, termed “variance-sensitive provisioning” (Ydenberg 1994; Ydenberg et al. 2007; Westneat et al. 2013, 2017).

Studies assessing variance-sensitive provisioning behavior are limited, and the few studies that have explicitly evaluated support for variance-sensitive provisioning have generated mixed support (Moore 2002; Westneat et al. 2013, 2017; Mathot et al. 2017). Further, within studies, support for variance-sensitive provisioning often differs across study years. For example, Mathot et al. (2017) observed patterns in residual variance in inter-visit intervals (i.e., the interval between two successive provisioning visits, or IVIs) which were consistent with variance-sensitive provisioning in a year with lower temperatures and more frequent rainfall (a so-called “bad year”) but not in a warmer, drier (“good”) year. Two other studies have also found year-specific support for variance sensitive provisioning (Moore 2002; Westneat et al. 2017). Based on these observations, it has been suggested that adoption of tactics to cope with increased brood demand is hierarchical wherein variance-sensitive provisioning is adopted as a last-resort. In other words, variance-sensitivity is adopted as a response only after increasing provisioning rate and altering prey selectivity have been exhausted as options in years with unfavorable provisioning conditions (Moore 2002; Mathot et al. 2017).

**Figure 2:**
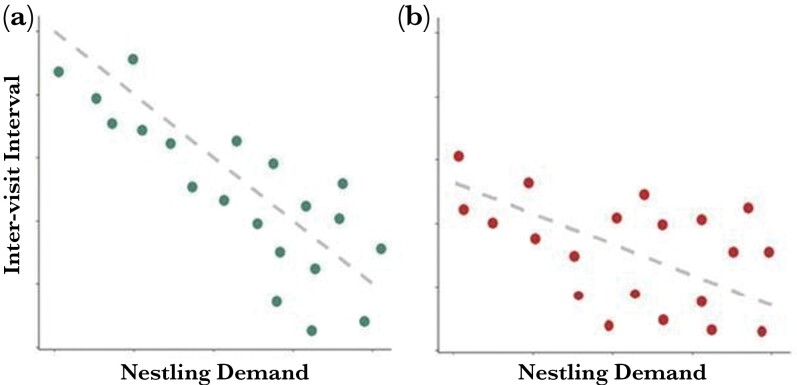
A schematic representing the predictions from the hierarchical model of provisioning behavior. While in all years it is expected that residual variance will increase with nestling demand (a) Years with high intercepts for inter-visit-interval (i.e., low parental effort) will also have steep reaction norm slopes in response to increasing nestling demand (increasing brood size or chick age) and low residual variance relative to (b) years with low intercepts (i.e., high parental effort) which will have shallow reaction norm slopes and high residual variance.

Parents should demonstrate plasticity in provisioning behavior within the constraints of year-specific conditions. Parents experiencing favorable foraging conditions are not expected to be working at their maximum level. Therefore, as brood demand increases (e.g., with increasing nestling age), parents should have scope to increase provisioning rate with increasing nestling demand either by allocating additional energy to provisioning behavior or by expanding diet breadth before switching to variance prone provisioning strategies. However, in years with unfavorable conditions, for example, years with inclement weather and/or low prey availability, parents may already be working near their maximum limits while including non-preferred prey in the diet. Thus, in unfavorable years, parents may have little scope to increase delivery rates to their young to satisfy increasing demand as nestlings age and may be expected to adopt variance sensitive provisioning tactics sooner. Thus, while it is expected that the use of variance-sensitive provisioning tactics should increase with increasing demand in all years, the point where this tactic is adopted is expected to come earlier with respect to nestling age (a proxy of brood demand) when conditions are unfavorable (Figure 2). To test this idea, assessment of individual reaction norms of provisioning behavior is required across both the duration of the period of parental care and across a range of environmental conditions (i.e., across multiple years). In particular, the hierarchical model of parental response to increased brood demand predicts specific patterns of covariance between provisioning to an average sized brood, that is, individual reaction norm intercept, change in provisioning, that is, slope, and variance in provisioning across years with lower year-specific intercepts (i.e., higher effort indicated by lower inter-visit interval) being associated with lower year-specific reaction norm slopes and higher year-specific variance (Figure 2).

There have only been four studies of variance-sensitive provisioning to date; three in passerines (Westneat et al. 2011, 2017; Mathot et al. 2017), one in terns (Moore 2002). However, raptors are also amenable to studies of variance-sensitive provisioning owing to their typically broad range of prey delivery options. Here, we studied provisioning behavior in Arctic-breeding Peregrine falcons (*Falco peregrinus tundrius).* We investigated support for patterns of covariance predicted by the hierarchical model of parental provisioning. To do this, we collected provisioning data from 99 nests over a 7-year period encompassing a range of conditions including wide variation in average seasonal temperatures, precipitation, and prey availability (see Hawkshaw et al. 2021). Our research addressed three questions. First, do provisioning rate and variance in provisioning rate increase with increasing nestling demand (primarily nestling age), which would be consistent with adoption of variance-sensitive provisioning with increasing brood demand? Second, do peregrines exhibit across-year differences in mean provisioning rates (i.e., intercept), adjustment in provisioning effort (i.e., plasticity or slope) with increasing nestling age, and variance in provisioning effort? Third, do intercept, slope, and variance in individual reaction norms covary as predicted by the hierarchical model of provisioning responses (Figure 2)? Specifically, we predicted that in years where parents are already provisioning at a high rate (low intercept for IVI) and have little scope to increase provisioning effort (shallow slope; small decrease in IVI), parents will adopt variance-sensitive provisioning behavior sooner (high variance in IVI) compared to years where parents are not working as hard (i.e., high IVI). This would result in negative among-year covariance between intercept and slope, intercept and variance, and a positive covariance between slope and variance.

## METHODS

### Study population and site

This study was conducted in a population of Peregrine falcons breeding close to Rankin Inlet, on the western shore of Hudson Bay, Nunavut (62.811998, -92.094354). The area is characterized primarily by tundra with numerous rocky outcrops, suitable for cliff nesting, across both coastal and inland areas. Further details on site-specific geological information, and a description of vegetation cover can be found in Court et al. (1988). Peregrines are cliff-nesting and nests at this study location are distributed across mainland, coastal, and island sites. The number of active nests varies between years, ranging from 16 to 30. This resulted in a total of 160 historically active nests being routinely visited across the 7-year duration of this study. Peregrines in our study area lay clutches of 2–4 eggs (Ratcliffe 1962) between early and late June, followed by incubation for approximately 32 days (with variability of 1–2 days, e.g., due to delayed hatching) (Burnham 1983; Anctil et al. 2013). Asynchronous hatching occurs in July, resulting in 1–4 nestlings (Ratcliffe 1962). The average hatch date for our study period was July 14th.

While data collection from nest sites is not without challenges (i.e., difficulties accessing nest sites and placing cameras), Peregrines are a generalist predator with a highly flexible prey range, including mammals, birds, and waterfowl. Our study population in Rankin Inlet has a particularly high contribution of mammalian prey in the diet (up to 1/3) compared to other peregrine populations that tend to be more specialized on birds (Bradley and Oliphant 1991; Ratcliffe 1993; Dawson 2011). This dietary flexibility makes them a good study organism for assessing variability in provisioning decisions. Although raptor species typically exhibit lower provisioning rates than passerines (i.e., visits where food is delivered to offspring), this can be offset by monitoring provisioning decisions for longer amounts of time (e.g., over days instead of over hours). In addition, our study area in Rankin Inlet, Nunavut is located within the Canadian Arctic, an area which experiences large inter-annual fluctuations in environmental conditions, that are likely to generate significant differences in foraging conditions experienced by provisioning adults across years.

### Data collection and processing

#### Life-history data

Historical nest sites were surveyed by ATV and snowmobile in May of each year, as Peregrines began to arrive at the breeding site. Sites were surveyed until enough nests had full clutches that continuing to survey unoccupied sites became unfeasible given constraints generated by limited availability of researchers in the field (typically late–June). Catching and banding of adults occurred between May and June using toe-snare methods outlined in the Wildlife Animal Care Committee Class Protocol #001—Raptor Collection for Falconry, provided by the Government of Alberta. Due to variation in catching effort, catching success, and logistical constraints, approximately 50% of adults in the breeding population each year are banded. This meant that we were unable to track individuals longitudinally across the study; either because they were unbanded in all years or were unbanded in some years before being banded.

We placed motion sensitive cameras (RECONXY models PC800, PC85, HC600, Ultrafire) attached to wooden stands within 1 m of all active nests, once located. All cameras were infrared enabled allowing images to be recorded during periods of low light. Cameras were set to record three images each time motion was detected, with 3 s between consecutive images, followed by a quiet period of 5–15 s during which the camera did not respond to motion triggers. In most years, cameras were also set to record time-lapse images. A single photo was taken at each time-lapse interval. A summary of camera settings for each year are provided in the electronic supplementary material (Supplementary Table S1).

We routinely visited occupied nests every 5–8 days (environmental conditions permitting). Once hatched, nestlings were marked on their upper right legs using non-toxic markers to allow individuals to be monitored throughout the season. The first hatched nestling was marked with red, and if applicable the next hatched with blue, next green, and the fourth was left unmarked. Using an electronic scale, we weighed individually marked nestlings at each visit and replaced camera batteries and memory cards as required. We also conducted 2-min focal observations of adults while at the nest site, as part of a separate study. Hatch dates were determined from nest camera images, from which nestling age was calculated for the first hatched nestling.

#### Provisioning data

We extracted provisioning data from time-stamped nest camera images from a total of 146 nests. In 2013, the hard drive on which the photos from 14 nests were stored was lost in the field, and we were only able to obtain provisioning data for 12 of 26 nests that had been monitored that year. Since we could only reliably score provisioning events that occurred within the camera frame, we restricted our data set to the period before the earliest age at which nestlings could move out of the nest scrape which we determined to be at nestling age 13 days based on observations of next camera images (R.A.M., personal observation). Thus, our analyses of provisioning data were restricted to the first 12 days after hatching.

We recorded the start and end time for each provisioning event. From this we calculated the interval between successive provisioning events calculated as the period between the start time of consecutive feeding visits, termed inter-visit interval, or IVI. We chose to use inter-visit intervals rather than the number of deliveries per day, as this provided multiple data points per day, improving our power to model variance. Although we were usually able to identify the sex of the parent that delivered the prey items to the nest (parental sex identified in 3915 of 5005 nest visits), we considered provisioning at the level of the pair of parents when calculating IVIs (in minutes). In other words, the time between two successive prey deliveries was used to calculate IVI regardless of whether the prey items were delivered by the male parent or the female parent, or a combination of the two. We felt this approach was justified because peregrines exhibit a division of labor, with males doing most of the hunting, and females doing most of the delivery to young (Olsen et al. 1998 and references therein). This was also the case in our dataset. In the first 12 days post-hatch, nestlings were fed almost exclusively by the female (*N* = 3873 visits out of 3915 visits where the parental sex could be identified). Thus, prey deliveries by females reflect the combined effects of male hunting effort and female allocation decisions. Given that we did not have the resolution of data to determine what percentage of prey items were hunted by male versus female parents, we limit our analysis to the level of the provisioning pair to match the resolution of our data with respect to provisioning effort. On average, 50% of birds were unbanded in any given study year. We assumed that the identity of unbanded males or females at a given nest site did not change within years, such that the identity of the provisioning pair was captured by the unique combination of nest site and year. However, our use of the combination of nest-site and year as a proxy for provisioning pair identity means that the same combination of male and female may be regarded as a different pair in a different year. However, this would tend to make our estimates of the importance of “provisioning pair” conservative, and our model comparisons none-the-less revealed “provisioning pair” to be important (see Results).

We determined camera failures, meaning periods in which the cameras should have been capturing images but were not (e.g., due to dead batteries, full memory cards) for years with time-lapse settings based on intervals of time between images which were larger than the preset time-lapse setting. These were recorded as “fail” in the datasheet. There was a total error rate of <0.5%, based on 5 years of data where time-lapse settings were used (2013–2014; 2017–2019). For the 2 years without time-lapse intervals (2015 and 2016), we excluded outliers in IVI (i.e., intervals between feeding events which were too long to be biologically possible) based on the distribution of datapoints from all 7 years (Supplementary Figure 1). This corresponded to 9 datapoints in total. The excluded datapoints were those above 4000 min (i.e., almost 67 h, or 2.8 days); at this cut off, there was a clear gap in points, with the next longest (included) datapoint being 2671 minutes (1.9 days) (see Supplementary Figure S1). Periods of camera failure were accounted for in IVI calculations wherein if the previous or current row was a recorded as “fail” then the IVI was recorded as “NA.” As part of a separate study, a total of 47 nests across the 7 years were food supplemented. We excluded these nests from the current study, leaving a total of 99 nests for inclusion in analysis (2013, *n* = 11; 2014, *n* = 14; 2015, *n* = 10; 2016, *n* = 11; 2017, *n* = 19; 2018, *n* = 16; 2019, *n* = 18).

We recognized IVI is an integrated measure that can reflect changes in various aspects of parental behavior. For example, parents can alter their allocation of time to provisioning relative to other activities, work harder while provisioning, and/or change the selectivity of prey items hunted/delivered. Using IVIs as our measure of parental effort does not allow us to tease apart the contributions of different aspects of parental behavior on provisioning rates. However, apart from the logistical challenges of collecting these other types of data, IVI is most appropriate for understanding the consequences of parental provisioning decisions on offspring, as it is the combined effect of parental behaviors on IVIs that ultimately determine offspring intake rates.

Originally, we intended to extract additional provisioning-related information, including prey type, biomass delivered, and biomass remaining after consumption by offspring. However, we were unable to accurately determine this information at 30% (4637 out of 15,395) of the provisioning visits made across the 7-year study duration due to poor image quality, prey being partially obstructed from view, etc. This is a substantial fraction of missing data, and more importantly, missing data is likely to be non-random with respect to prey characteristics (e.g., small prey more difficult to score than large prey). Furthermore, our nest cameras collected images only of the scrape, and did not include images of the surrounding areas, for example, where prey may have been cached or processed prior to delivery. Thus, prey type and size data, which we were able to score from camera trap images, were incomplete and likely biased, and we therefore did not use it for further analysis. We did, however, note that Peregrines in our study provided at least 7683 small birds (passerines, shorebirds), 3430 mammals (ground squirrels, lemmings), and 1438 waterfowl (ducklings/goslings of various sizes) to offspring. Note, the total visits where prey type was scored (*N* = 12,551) exceed the number for which we had complete data (type + biomass: *N* = 10,758, see above) because we were able to score prey type but not biomass for *N* = 1793 visits.

### Statistical analysis

All models were run using the brms package (Bürkner 2017) in R (version 4.2.0). We followed Nussey et al. (2007) hierarchical approach to assess support for random effects of increasing complexity. In each model, IVI was modeled as a function of brood size and nestling age (fixed effects) and sigma (i.e., variance) was modeled as a function of nestling age and brood size. Year was included as a random effect. Starting from the described basic model, we then increased model complexity by adding random effects for nest site, provisioning pair (i.e., the specific combination of year and site), year-specific random slopes, as well as covariances between random effects.

Table 1 provides an overview of the models that were tested. We compared these models using leave one out cross validation with the “loo_compare” function from the “loo” package (Vehtari et al. 2017) (see Table 1 for output). The “elpd_diff” and “se_diff” values obtained provide an estimate of the difference in the expected log pointwise predictive density (elpd) and its standard error between models. A larger “elpd_diff” and smaller “se_diff” indicate that one model performs better than another, and a difference in “elpd” of 2 or more units is generally considered to be strong evidence in favor of one model over another (Vehtari et al. 2017).

**Table 1:** A table containing details of across-year models run for comparative purposes to first verify evidence for the existence of random effects for nest site, provisioning pair, year, year-specific random slopes and second to assess evidence of covariance between random effects. All models additionally contained a sigma model with nestling age and brood size as fixed effects, and year as a random effect. Differences between models in terms of “elpd” (and se) indicate that while the model which contained all random effects but did not estimate covariances performed slightly better than that which estimated covariance, there are negligible differences between the top three competing models

Model	Nest site	Pair	Year	Year (nestling age)	Year (brood size)	Sigma	Covariance	Elpd_diff (se_diff)	Rank
m1a						x		−134.8 (17.0)	8
m1b	x					x		−40.4 (10.1)	7
m1c		x				x		−3.7 (3.9)	6
m1d	x	x				x		−3.7 (3.7)	6
m1e	x	x	x			x		−2.5 (3.0)	5
m1f	x	x	x	x		x		0.0 (0.0)	1
m1g	x	x	x			x	x	−0.6 (3.1)	3
m1h	x	x	x	x		x	x	−0.2 (0.4)	2
m1i	x	x	x	x	x	x	x	−0.7 (0.5)	4

While we were interested in year-specific differences in constraints on provisioning behavior, we anticipated that the analyses described above, which analyze year-specific provisioning responses, could generate patterns consistent with our predictions, not necessarily because of within-individual responses (the hypothesized mechanism). For example, year-specific differences in the types of individuals which are successful within a given year could also result in the patterns of covariance outlined in Figure 2. To address this, we ran an additional model for comparative purposes, which was the same as the top-performing model from the previously described model set with two exceptions: 1) nestling age was nested within pair id, and 2) sigma (i.e., residual variance) was estimated across pairs (hereafter the “across pairs model”) rather than across years (hereafter the “across years model”). By running both models, we could evaluate whether patterns at the within-pair level were qualitatively similar to patterns at the among-year level, which would provide support for the interpretation that year-specific patterns arose via within-individual plasticity. Unless otherwise specified, results presented are those generated by the (top performing) across years model.

For all models, IVI was log-transformed prior to analysis to ensure that model residuals were normally distributed. On Day 0 of brood hatching (i.e., on the day the first nestling hatches) provisioning rates cannot be sensibly compared for a nestling that hatched in the morning versus in the evening, for example, and so nestling age 0 was excluded from the analysis. Further, nestling age was subsequently left zeroed (i.e., 1 was subtracted for each row of nestling age so that nestling Day 1 was coded as zero). This meant that model intercepts were estimated for day 1 post-hatching, the first day when provisioning behavior was analyzed. Additionally, brood size was centered so that the model intercept was estimated at the average number of nestlings. Further, to facilitate comparison between influence of fixed effects on IVI, we scaled (standardized) both nestling age and brood size by dividing values by 1 standard deviation.

We used the mode of estimated effects (β, σ, or ρ) and 95% credible intervals (CI) to evaluate support for each effect. Strong support for an effect is defined here as an effect with CIs which do not overlap zero. Moderate support is defined as CIs that overlapped zero by less than 15%. Posterior distribution which overlaps zero by 15% or less equates to over five times greater support for interpretation of an effect in the estimated direction relative to an effect in the opposite direction (Marsman and Wagenmakers 2017). If the estimated effect was approximately zero and CIs centered around zero, we interpret this as providing no support for the effect (also referred to as “strong support for no effect.”). When discussing the potential biological importance of a given observed effect, we use the estimated effect size on the observed scale for fixed effects (e.g., the effect of increasing nestling age in days on IVI in seconds). However, as variance estimates are unitless, we interpret the biological importance of random effects based on their relative contributions to total variance (i.e., proportion contribution). All results presented are from the top performing across-year model, unless otherwise specified.

## RESULTS

**Figure 3:**
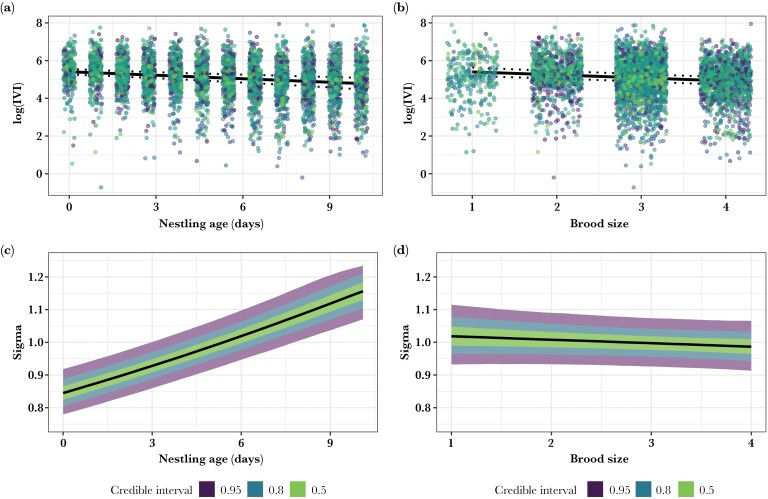
A figure displaying observations and model posterior predictions for (a) IVI across nestling age, (b) IVI across brood size, (c) standard deviation, σ (sigma: the root-square of variance) in IVI across nestling age, and (d) variance in IVI across brood size. Credible intervals are displayed for 0.95, 0.8, and 0.5. Plots were made using R packages “gghalves” and “ggplot2.”

At the population level, peregrines responded to increased brood demand (as indicated by nestling age and brood size) by reducing their provisioning inter-visit intervals (IVIs, i.e., provisioning at a higher rate). This was true across all study years. There was some support that parents decreased IVI to a greater extent with increasing nestling age (β_log(IVI)_ = −0.18, 95% CIs = −0.25, −0.11; Figure 3a) compared to increasing brood size (β_log(IVI)_ = −0.12, 95% CIs = −0.16, −0.07; Figure 3b), though the difference was not significant.

As predicted, we found strong support (based on CIs that did not overlap 0) that variance in logIVI increased with increasing nestling age (β_log(σ)_ = 0.08, 95% CIs = 0.06, 0.11; Figure 3c). Further, the increase in variance across ages is >2 fold greater than the variance within ages (see Figure 3c). However, contrary to our predictions, there was no evidence that variance in log(IVI) increases with increasing brood size (β_log(σ)_ = −0.01, 95% CIs = −0.03, 0.01; Figure 3d). This indicates that as nestling demand generated by increasing nestling age (but not number of nestlings) increased, parents demonstrated greater variability in provisioning effort (measured by IVI).

Our model comparisons revealed strong support for the presence of all the random effects considered: among-pair, among-site, and among-year differences in IVI intercept, among-year differences in reaction norm slope (plasticity), and among-year differences in residual variance (sigma) in IVI in response to increasing nestling age (Table 1). The variation in each of these random effects were of magnitudes that were likely to be biologically important. For example, the intercept in log-transformed inter-visit-interval (logIVI) was lowest in 2016 (β_log(IVI)_ = 5.31, 95% CI = 4.97, 5.59) and highest in 2013 (β_log(IVI)_ = 5.48, 95% CIs = 5.22, 5.84). This corresponded to time between the start of consecutive provisioning visits to an average-sized brood of approximately 3.4 h in 2016 compared with approximately 4 h in 2013. The relative change in IVI with increasing brood demand generated by increasing nestling age was greatest (steepest slope) in 2017 (β = −0.52, 95% CI = −0.88, −0.18) and smallest (shallowest) in 2013 (β_log(IVI)_ = −0.26, 95% CIs = −0.61, 0.13). This corresponds to a decrease in IVI of around 20 minutes from nestling age 1 to day 12 post-hatch in 2017, versus a decrease in IVI of 15 min in 2013. Variance in logIVI was greatest in 2016 (σ_(exp)_ = 0.85, 95% CIs = 0.62, 1.17) and smallest in 2014 (σ_(exp)_ = 0.63, 95% CIs = 0.45, 0.89). Even though pairs often used the same site across multiple years, among site differences in logIVI (β_log(IVI)_ = 0.14, 95% CIs = 0.02, 0.25) were lower than among pair differences (β_log(IVI)_ = 0.19, 95% CIs = 0.12, 0.26) suggesting that differences in logIVI are not just about territory quality, but that pair quality is a more important driver of variation in provisioning rates.

Our model comparison revealed that the top three models are within 2 units of difference in “elpd” (Table 1) and are, therefore, indiscernible (Vehtari et al. 2017). Specifically, models which additionally estimated covariances between random effects performed similarly to the model which estimated all random effects, but not their covariances. Thus, support for covariances is equivocal. Nonetheless, we report the estimated covariances to allow us to evaluate whether they are in qualitative agreement with the predictions outlined based on the hierarchical adoption of provisioning tactics (Figure 2).

We found weak evidence for a moderate negative correlation between intercept and variance in logIVI across the 7 study years (ρ = −0.25, 95% CIs = −0.91, 0.67; Supplementary Figure S2b) meaning that in years with a higher intercept (i.e., a longer time between consecutive provisioning visits to an average-sized brood) there was less variance in logIVI relative to years with a lower intercept. There was no support for a correlation between slope and variance (ρ = 0.04, 95% CIs = −0.71, 0.76; Supplementary Figure S2c) meaning that, contrary to our prediction, year-specific responses to increased brood demand were unrelated to year-specific variation in IVIs. We found a weak, positive correlation between intercept and slope (ρ = 0.22, 95% CIs= = −0.71, 0.92; Supplementary Figure S2a) such that higher intercepts (longer time between consecutive provisioning visits to an average sized brood) resulted in shallower slopes (smaller reduction in time between consecutive provisioning visits), and vice versa. Again, wide confidence intervals around the estimate prevent us from drawing strong conclusions regarding the precise magnitude of the correlation.

Results regarding correlations between intercept and variance, and slope and variance were qualitatively similar for both the across years and across pairs model (for across pair model, intercept vs. variance: ρ = −0.52, 95% CIs −0.90, −0.07, slope vs. variance: ρ = 0.19, 95% CIs = −0.22, 0.58). However, we found weak support for a moderately negative correlation between intercept and slope in the across pairs model (ρ = −0.25, 95% CIs = −0.64, 0.38, unlike in the across years model). Thus, the results from the across-pair model suggest that higher intercepts result in steeper slopes, that is, a longer time between consecutive provisioning visits to an average-sized brood results in comparatively larger reductions in IVIs as nestling age increases. However, again, wide confidence intervals limit the inference we can draw from current data.

## DISCUSSION

The hierarchical model of provisioning proposes that parents should make strategic, sequential, use of three provisioning tactics as offspring demand increases. Initially, parents may increase their own energy expenditure to meet additional offspring needs, typically leading to an increased provisioning rate (Sanz and Tinbergen 1999; Magrath et al. 2007; Steen et al. 2012; Bowers et al. 2014; Laczi et al. 2017). In response to further increase in demand, parents may expand the provisioned diet breadth (e.g., Grundel and Dahlsten 1991; Wright et al. 1998; Małgorzata 2004; Radford 2008; Wiebe and Slagsvold 2015). Finally, parents may adopt variance-sensitive provisioning once these two other options have been exhausted (Westneat et al. 2011, 2017; Mathot et al. 2017). If parents adopt different strategies for managing increased brood demand hierarchically, as outlined above, then we predicted that parents should increase their provisioning rate with increasing brood demand (i.e., reduce IVI) and demonstrate increased variance in IVI with increasing nestling demand. More specifically, we predicted that parents provisioning at a higher rate (lower IVI) to an average sized brood should have reduced scope to increase provisioning (reduce IVI) as demand increases, and therefore, adopt variance-sensitive provisioning strategies sooner, resulting in higher residual variance in provisioning. We evaluated support for these predicted patterns of residual variance and covariance in an Arctic breeding population of Peregrine falcons. We found strong support that peregrines decrease provisioning IVIs with increased nestling age and increasing brood size. We also found strong support for an increase in residual variance in IVI with increasing brood demand generated by increasing nestling age, but not increasing brood size. However, we did not find strong support for predictions based on the hierarchical adoption of tactics for coping with increased brood demand. Support for covariances between year-specific provisioning effort, provisioning reaction norms, and residual variance in provisioning were equivocal, and not always in the predicted direction. We discuss the biological insights revealed by our approach, as well as the limitations of the current study, including the challenge of modeling heterogeneous residual variance, particularly using observational field data. Finally, we suggest how researchers aiming to address similar questions may overcome these challenges.

As predicted based on parental investment theory (Emlen 1966; MacArthur and Pianka 1966; Trivers 1972), across years, provisioning adult peregrines consistently responded to increased nestling demand, as inferred by nestling age and nestling number, by reducing IVI. At least one earlier study in peregrines reported that parental response to increasing brood demand via increased nestling age was greater compared with parental response to increased brood demand via increased brood sizes (Olsen et al. 1998). Although our results are qualitatively in agreement, the difference in parental reaction norm to increased nestling age versus increased brood size was not statistically significant in our study. However, an important distinction between our study and that of Olsen et al. (1998) is that they distinguished between prey deliveries by male versus female parents and found a different response to nestling age versus brood size in males only. In our study, we were not able to differentiate male and female provisioning effort and used a pair-level measure of provisioning.

We found that provisioning pairs (i.e., the specific combination of year and nest site) had a greater influence on plasticity in IVI than territory (i.e., nest site), which is consistent with both existing theory and empirical results. At our research site, for example, earlier empirical work by Bradley and Oliphant (1991) found that Peregrines hunt not only small birds but around a third of their total consumed biomass is mammalian (microtines, primarily Arctic Ground Squirrels [*Urocitellus parryii*] and Lemmings [various *Dicrostonyx* species]); a proportion markedly higher even than for other studied Peregrine populations (Bradley and Oliphant 1991 and references therein). Thus, while peregrines are highly territorial and differences in prey availability across territories can be significant (e.g., Sokolov et al. 2014), we suggest that across nest sites in our study population, differences in territory quality may be less important as parents may have access to comparable total prey availabilities due to the relatively large diet breadth reported previously in our population (Bradley and Oliphant 1991). On the other hand, the impact of pair quality on parental care (e.g., provisioning behavior) is widely reported in existing literature. For example, older and more experienced parents typically raise more offspring and/or offspring in better condition (Curio 1983; Pugesek 1995; Angelier et al. 2006; Pittet et al. 2012), as least up to a certain point, where parental age effects may either plateau or even reverse in the case of senescence (Zabala and Zuberogoitia 2015).

We predicted an increase in residual variance in IVI with increasing brood demand because provisioning adults should be increasingly variance prone as offspring demand increases beyond what they can satisfy by expending more energy and/or reducing prey selectivity (Mathot et al. 2017). We found strong support that parent IVIs became more variable as nestlings aged. This result is notable because mean and variance are normally positively correlated (Cohen and Xu 2015). Thus, the null expectation would be that the decrease in mean IVI with increasing nestling age would coincide with a decrease in variance in IVI. We interpret the observed increase in the residual variance in IVI with increasing nestling age as a strategic shift toward more variable foraging options in response to increased nestling demand. However, at least three alternative explanations are possible. First, higher residual IVI with increasing nestling age may result from depletion of local food as the season progresses (Lima and Dill 1990), making food discoveries less predictable. We suggest this is unlikely because the increased variance in IVI was coincident with a decrease in mean IVI, indicating that parents were returning with food more quickly with increasing nestling age.

Alternatively, the decrease in IVI and coincident increase in residual variance in IVI with increasing nestling age may reflect an increased availability of prey (both quantity and type) as the potential prey increase in abundance on the landscape (e.g., due to production of young by breeding birds). Indeed, a study monitoring changes in avian abundance and distribution in our study area reported increases in the abundance of on shorebirds and sandhill cranes (but decreases or no change in other groups) (Hawkshaw et al. 2021). However, these changes in avian abundance were modest over the circa 120-day monitoring period and may have been confounded by changes in detectability (Hawkshaw et al. 2021). The changes in IVI (mean and variance) reported here occurred within 12-day periods (at each nest), which is unlikely to be sufficient for large-scale changes in prey availability across the landscape due to production of young.

A third possibility is that as nestlings age, the type and size of prey they can consume expands, for example, due to larger bill gape or reduced digestive constraints. In an earlier study, Cade (1982) found the proportion of mammals in the diet of Peregrine nestlings decreased as nestling age increased, while the proportion of small birds increased from around 10–90% over the first 4 weeks post-hatch. However, the study did not report on how much of this change occurred in the first 12 days post-hatch, which is the period over which decreasing mean IVI and increasing variance in IVI were observed in our study. Drent and Daan (1980) found that the gape size of several bird species increased rapidly during the first few weeks of life, allowing them to consume larger prey items during each provisioning visit. Although gape size increases in Peregrine nestlings, Peregrine nestlings are exclusively fed by parents until beyond 12 days post-hatch (i.e., the period examined in this study). Thus, parents can provision large prey items to nestlings with small gape sizes by tearing off appropriately sized pieces and consuming the remainder of prey themselves or caching it. Therefore, it is unclear whether age-related changes in gape size should change the type or size of prey provisioned by parents. We cannot rule out that increasing diet breath (type and/or size) with increasing nestling age contributed to the finding that mean IVI decreased while variance in IVI increased across nestling age. We suggest that studies that directly investigate age-related changes in prey type and size provisioned to nestling peregrines are warranted.

Interestingly, we did not find any support for parents increasing variance in IVI with increasing brood size. There are at least two potential explanations for this result. First, brood size might vary with parental quality such that better quality parents are more likely to have larger broods. In this case, larger brood sizes would not be expected to be associated with higher variance in provisioning because high quality parents are better equipped to cope with increased brood demand, and therefore, all else being equal, would be expected to resort to variance sensitive provisioning at higher levels of brood demand, including brood size. To properly evaluate this possibility would require experimental manipulations of brood size. Alternatively, if the observed pattern of decreasing mean IVI and increasing variance in IVI across nestling age is the result of an increase in the breadth of suitable prey, we would not expect to see a change in variance in IVI with larger brood sizes. This is because, unlike nestling age, larger brood sizes would not correlate with increased availability of suitable prey.

We found strong support for the inclusion of random effects for among-pair and among-site differences in IVI, and among-year differences in intercept (IVI to an average-sized brood), reaction norm slope (plasticity), and residual variance in IVI in response to increasing nestling age. We tested among-year correlations between intercept, slope and variance in IVI based on earlier studies that proposed that tactics for responding to increased brood demand might be adopted hierarchically (sequentially), with variance-sensitive responses adopted as a last resort. Although, the qualitative patterns of covariance did not always align with our predictions (Figure 2), the high degree of uncertainty in the covariance estimates (i.e., broad 95% CIs) means that we cannot draw strong conclusions with the current data.

Overall, our study provides mixed support for the notion that peregrines adopt variance-sensitive provisioning decisions to cope with increased brood demand. Although we found strong support for increased residual variance across nestling age, we likely lacked statistical power to estimate the covariances between random intercept, random slope and year-specific residual variance despite the fact that our analyses included over 5000 unique provisioning visits from 99 nests at 51 nest sites over 7 years. This highlights the challenge of modeling heterogeneous residual variances, which are known to be data-hungry analyses (Cleasby and Nakagawa 2011). In recent years, there has been a push in behavioral ecology to recognize the prevalence of heteroscedasticity in datasets (e.g., (Nakagawa et al. 2007; Westneat et al. 2013), which has been under-reported despite being a source of important information about the biological processes being investigated (Cleasby and Nakagawa 2011; Westneat et al. 2015). Our results emphasize both challenges (data requirements) and opportunities (novel insights) that can be afforded by quantifying heterogeneous residual variance. The finding that residual variance in provisioning IVIs increases with increasing nestling age despite decreasing mean IVI hints at two (non-exclusive) mechanisms that may underlie age-related shifts in parental care decisions; variance sensitivity and/or age-related changes in diet.

In addition to the need for larger sample sizes, we suggest that across-year comparison of IVI alone may have been insufficient to fully assess patterns in provisioning in our study because it does not account for inter- or intra-annual variation in provisioned prey type or size, which may be substantial (Hawkshaw et al. 2021). All else being equal, parents exert more energy to reduce IVI. However, in years with an abundance of preferred prey, for example, it may be easier for parents to hunt and thus exhibit lower IVIs without expending additional energy. Alternatively, parents may opt to bring larger prey per visit instead of reducing IVI as demand increases. For example, peregrines breeding near Canberra, Australia, maintained comparable nestling growth rates in both control and enlarged broods by increasing the size (and biomass) of delivered prey items to experimentally enlarged broods (Olsen and Tucker 2003). Several other studies report Peregrines increasing mass of delivered prey items instead of provisioning rate (Olsen and Tucker 2003; Palmer et al. 2004; Dawson et al. 2011; but see Zuberogoitia et al. 2013). Provisioning larger prey items increases the likelihood of offspring survival (Dawson et al. 2011). We suggest that future studies evaluating hierarchical models of provisioning either focus on systems in which there is little scope for inter-annual variation in prey type or quantify prey type and size in addition to IVI. Unfortunately, we were unable to do this in the present study as we were unable to identify prey type and/or size in approximately 30% of visits (4637 out of 15,395 visits). As the success rate of prey identification varied across nests (due to camera placement), and likely due to prey type/size (e.g., larger prey easier to identify), we did not feel confident that our sampling of prey type/size was unbiased, and therefore, did not attempt to include analyses of prey type/size in the present study.

Additionally, the observed patterns of shifts in IVI may arise through any combination of tactics, for example, allocation of time to provisioning behavior and/or hunting, quantity of prey consumed by the parents themselves versus delivered to offspring, selectivity of prey items hunted and delivered, and time spent processing hunted prey items prior to delivery. However, based on data collected in our study (nest camera images of exclusively the scrape), it was not possible to assess correlations between specific provisioning behaviors beyond IVI and the corresponding changes in IVI, to provide deeper insights into which tactics may be most prevalent and how they impact temporal patterns of provisioning as nestling demand increases. Future work is needed to ascertain the specific tactic(s) responsible for the patterns observed in this study.

Our study provides insights into the plasticity of parental investment decisions in Peregrine falcons and raises new questions for future research. While we did not find strong support for the predicted covariances between provisioning intercept, plasticity and variance, modeling heterogeneous residual variance and covariances in intercept, slope, and variance are data-hungry analyses. Future work aimed at testing hierarchical provisioning decisions will require larger sample sizes. Nonetheless, our analyses did yield some novel insights. Consistent with a large body of work on parental investment theory, our results show that provisioning adult peregrines respond to increased nestling demand, both in terms of nestling age and nestling number, by reducing IVI. However, we also found that parents increase residual variance in IVI as a function of increasing nestling age, but not in response to increasing nestling number. This finding is consistent with a strategic shift in behavior toward more variable foraging options with increased nestling demand as nestlings age. Further studies are needed that simultaneously track variation in provisioning rates and the size and type of prey provisioned to allow for strong inference on the relative importance of variance sensitivity versus increased diet breadth (size and/or type) with increasing nestling age in generating the age-related patterns in parental provisioning reported here.

## Supplementary Material

arad103_suppl_Supplementary_Figures

arad103_suppl_Supplementary_Tables

## Data Availability

Analyses reported in this article can be reproduced using the data and code provided by McKinnon et al. (2023).
