# Supplementary material for: Peregrine falcons shift mean and variance in provisioning in response to increasing brood demand
Source: Behav Ecol. 2023 Dec 22;35(1):arad103. doi: 10.1093/beheco/arad103 (PMC10746350; doi:10.1093/beheco/arad103)
Supplement: arad103_suppl_Supplementary_Figures [file arad103_suppl_supplementary_figures.docx]

Electronic Supplementary Materials (ESM):

**
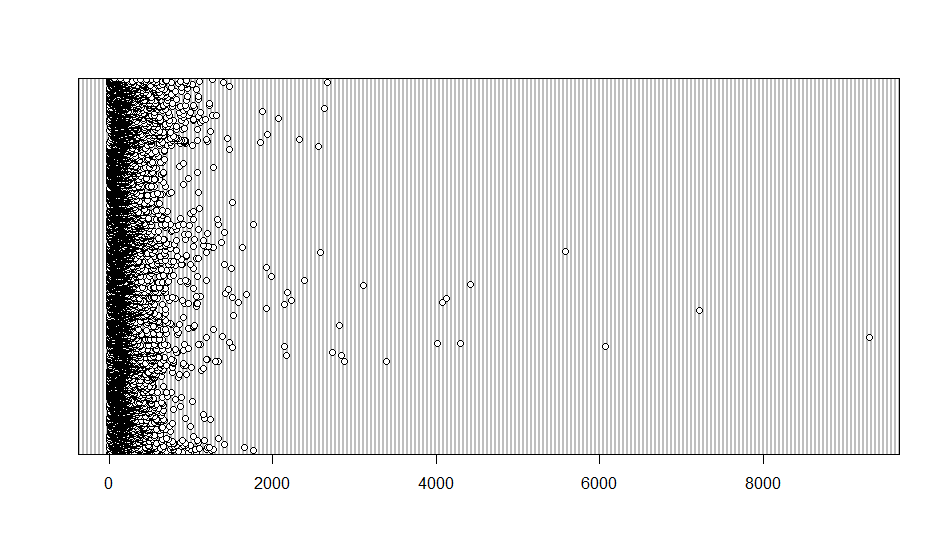
**

**Figure S1**

A dotchart showing the distribution of IVI (untransformed) across all 7 study years. A cut-off of 4000 minutes was set when determining outliers, resulting in the exclusion of 9 datapoints.

**
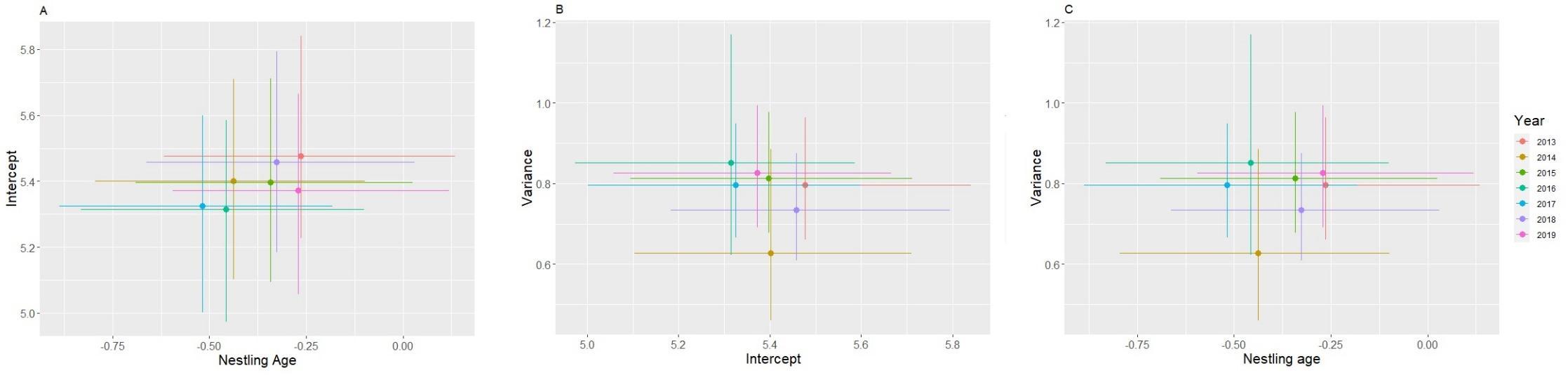
**

**Figure S2**

Figures showing the covariances between intercept, slope and variance in logIVI across 7 study years (means and 95% credible intervals are reported). (a) There was a weak, positive covariance between intercept and slope generated by increasing nestling age, (b) a moderate, negative covariance between intercept and variance, and (c) no evidence of covariance between slope and variance.
