# Supplementary material for: Peregrine falcons shift mean and variance in provisioning in response to increasing brood demand
Source: Behav Ecol. 2023 Dec 22;35(1):arad103. doi: 10.1093/beheco/arad103 (PMC10746350; doi:10.1093/beheco/arad103)
Supplement: arad103_suppl_Supplementary_Tables [file arad103_suppl_supplementary_tables.docx]

**Table S1**

A summary of camera settings (time lapse, motion sensitivity, and camera model used) organized by site and year.

| **Site** | **Year** | **Time Lapse Setting** | **Motion Sensitivity** | **Camera Model** |
| --- | --- | --- | --- | --- |
| 4 | 2015 | 15 | 75 | PC85 RAPIDFIRE PRO |
| 4 | 2015 | no timelapse | 100 | PC85 RAPIDFIRE PRO |
| 8 | 2015 | 15 | 100 | PC85 RAPIDFIRE PRO |
| 8 | 2015 | no timelapse | 100 | PC85 RAPIDFIRE PRO |
| 12 | 2015 | 15 | 75 | PC85 RAPIDFIRE PRO |
| 12 | 2015 | no timelapse | 100 | PC85 RAPIDFIRE PRO |
| 19 | 2015 | 15 | 75 | PC85 RAPIDFIRE PRO |
| 19 | 2015 | no timelapse | 75 | PC85 RAPIDFIRE PRO |
| 19 | 2015 | no timelapse | 100 | PC85 RAPIDFIRE PRO |
| 20 | 2015 | 15 | 75 | PC85 RAPIDFIRE PRO |
| 20 | 2015 | no timelapse | 100 | PC85 RAPIDFIRE PRO |
| 30 | 2015 | 15 | 75 | PC85 RAPIDFIRE PRO |
| 30 | 2015 | no timelapse | 100 | PC85 RAPIDFIRE PRO |
| 33 | 2015 | 15 | 75 | PC85 RAPIDFIRE PRO |
| 33 | 2015 | no timelapse | 100 | PC85 RAPIDFIRE PRO |
| 35 | 2015 | no timelapse | 100 | PC85 RAPIDFIRE PRO |
| 39 | 2015 | 15 | 75 | PC85 RAPIDFIRE PRO |
| 39 | 2015 | no timelapse | 100 | PC85 RAPIDFIRE PRO |
| 53 | 2015 | no timelapse | 100 | PC85 RAPIDFIRE PRO |
| 59 | 2015 | 15 | 75 | PC85 RAPIDFIRE PRO |
| 59 | 2015 | no timelapse | 100 | PC85 RAPIDFIRE PRO |
| 72 | 2015 | 15 | 75 | PC85 RAPIDFIRE PRO |
| 72 | 2015 | no timelapse | 100 | PC85 RAPIDFIRE PRO |
| 75 | 2015 | no timelapse | 100 | PC85 RAPIDFIRE PRO |
| 89 | 2015 | no timelapse | 100 | PC85 RAPIDFIRE PRO |
| 95 | 2015 | no timelapse | 75 | PC85 RAPIDFIRE PRO |
| 119 | 2015 | 15 | 75 | PC85 RAPIDFIRE PRO |
| 119 | 2015 | no timelapse | 100 | PC85 RAPIDFIRE PRO |
| 130 | 2015 | 15 | 75 | PC85 RAPIDFIRE PRO |
| 130 | 2015 | no timelapse | 100 | PC85 RAPIDFIRE PRO |
| 1 | 2016 | no timelapse | 100 | PC85 RAPIDFIRE PRO |
| 3 | 2016 | no timelapse | 100 | HC600 HYPERFIRE |
| 4 | 2016 | no timelapse | 75 | PC85 RAPIDFIRE PRO |
| 8 | 2016 | no timelapse | 100 | PC85 RAPIDFIRE PRO |
| 12 | 2016 | no timelapse | 100 | PC85 RAPIDFIRE PRO |
| 19 | 2016 | no timelapse | 100 | PC85 RAPIDFIRE PRO |
| 19 | 2016 | no timelapse | 75 | PC85 RAPIDFIRE PRO |
| 23 | 2016 | no timelapse | 100 | HC600 HYPERFIRE |
| 30 | 2016 | no timelapse | 100 | PC85 RAPIDFIRE PRO |
| 31 | 2016 | no timelapse | 100 | PC800 PROFESSIONAL |
| 35 | 2016 | no timelapse | 50 | PC85 RAPIDFIRE PRO |
| 37 | 2016 | no timelapse | 100 | PC800 PROFESSIONAL |
| 41 | 2016 | no timelapse | 100 | PC800 PROFESSIONAL |
| 42 | 2016 | no timelapse | 100 | PC85 RAPIDFIRE PRO |
| 51 | 2016 | no timelapse | 100 | PC85 RAPIDFIRE PRO |
| 53 | 2016 | no timelapse | 100 | PC85 RAPIDFIRE PRO |
| 58 | 2016 | no timelapse | 100 | PC85 RAPIDFIRE PRO |
| 59 | 2016 | no timelapse | 100 | PC800 PROFESSIONAL |
| 63 | 2016 | no timelapse | 100 | PC85 RAPIDFIRE PRO |
| 63 | 2016 | no timelapse | 75 | PC85 RAPIDFIRE PRO |
| 75 | 2016 | no timelapse | 100 | PC85 RAPIDFIRE PRO |
| 77 | 2016 | no timelapse | 100 | PC85 RAPIDFIRE PRO |
| 77 | 2016 | no timelapse | 75 | PC85 RAPIDFIRE PRO |
| 78 | 2016 | no timelapse | 100 | PC85 RAPIDFIRE PRO |
| 78 | 2016 | no timelapse | 75 | PC85 RAPIDFIRE PRO |
| 85 | 2016 | no timelapse | 100 | PC85 RAPIDFIRE PRO |
| 85 | 2016 | no timelapse | 50 | PC85 RAPIDFIRE PRO |
| 89 | 2016 | no timelapse | 100 | PC800 PROFESSIONAL |
| 95 | 2016 | no timelapse | 100 | PC800 PROFESSIONAL |
| 97 | 2016 | no timelapse | 100 | PC85 RAPIDFIRE PRO |
| 97 | 2016 | no timelapse | 50 | PC85 RAPIDFIRE PRO |
| 100 | 2016 | no timelapse | 100 | PC85 RAPIDFIRE PRO |
| 110 | 2016 | no timelapse | 100 | PC85 RAPIDFIRE PRO |
| 130 | 2016 | no timelapse | 100 | PC85 RAPIDFIRE PRO |
| 145 | 2016 | no timelapse | 75 | PC85 RAPIDFIRE PRO |
| 147 | 2016 | no timelapse | 100 | PC85 RAPIDFIRE PRO |
| 1 | 2017 | 15 | 75 | PC85 RAPIDFIRE PRO |
| 1 | 2017 | 15 |  | PC85 RAPIDFIRE PRO |
| 1 | 2017 | 15 | 100 | PC85 RAPIDFIRE PRO |
| 5 | 2017 | 15 | 100 | PC85 RAPIDFIRE PRO |
| 8 | 2017 | 15 | 100 | PC85 RAPIDFIRE PRO |
| 8 | 2017 | 15 | 75 | PC85 RAPIDFIRE PRO |
| 12 | 2017 | unknown | unknown | PC800 PROFESSIONAL |
| 18 | 2017 | 15 | 75 | PC85 RAPIDFIRE PRO |
| 18 | 2017 | 15 | 100 | PC85 RAPIDFIRE PRO |
| 19 | 2017 | unknown | unknown | PC800 PROFESSIONAL |
| 22 | 2017 | unknown | unknown | PC800 PROFESSIONAL |
| 23 | 2017 | unknown | unknown | PC800 PROFESSIONAL |
| 28 | 2017 | unknown | unknown | PC800 PROFESSIONAL |
| 30 | 2017 | unknown | unknown | PC800 PROFESSIONAL |
| 31 | 2017 | unknown | unknown | HC600 HYPERFIRE |
| 33 | 2017 | 15 | 75 | PC85 RAPIDFIRE PRO |
| 33 | 2017 | 15 | 100 | PC85 RAPIDFIRE PRO |
| 42 | 2017 | unknown | unknown | PC800 PROFESSIONAL |
| 51 | 2017 | 15 | 100 | PC85 RAPIDFIRE PRO |
| 53 | 2017 | unknown | unknown | PC800 PROFESSIONAL |
| 58 | 2017 | 15 | 100 | PC85 RAPIDFIRE PRO |
| 59 | 2017 | 15 | 75 | PC85 RAPIDFIRE PRO |
| 59 | 2017 | 15 | 100 | PC85 RAPIDFIRE PRO |
| 63 | 2017 | 15 | 100 | PC85 RAPIDFIRE PRO |
| 75 | 2017 | 15 | 100 | PC85 RAPIDFIRE PRO |
| 77 | 2017 | 15 | 75 | PC85 RAPIDFIRE PRO |
| 77 | 2017 | 15 | 100 | PC85 RAPIDFIRE PRO |
| 78 | 2017 | unknown | unknown | HC600 HYPERFIRE |
| 85 | 2017 | 15 | 100 | PC85 RAPIDFIRE PRO |
| 89 | 2017 | 15 | 75 | PC85 RAPIDFIRE PRO |
| 89 | 2017 | 15 | 100 | PC85 RAPIDFIRE PRO |
| 95 | 2017 | 15 | 75 | PC85 RAPIDFIRE PRO |
| 95 | 2017 | 15 | 100 | PC85 RAPIDFIRE PRO |
| 97 | 2017 | unknown | unknown | PC800 PROFESSIONAL |
| 98 | 2017 | unknown | unknown | PC800 PROFESSIONAL |
| 100 | 2017 | unknown | unknown | PC800 PROFESSIONAL |
| 145 | 2017 | 15 | 75 | PC85 RAPIDFIRE PRO |
| 145 | 2017 | 15 | 100 | PC85 RAPIDFIRE PRO |
| 147 | 2017 | unknown | unknown | PC800 PROFESSIONAL |
| 1 | 2018 | 5 | unknown | PC85 RAPIDFIRE PRO |
| 3 | 2018 | 15 | unknown | PC85 RAPIDFIRE PRO |
| 3 | 2018 | 5 | unknown | PC85 RAPIDFIRE PRO |
| 18 | 2018 | 5 | unknown | PC85 RAPIDFIRE PRO |
| 22 | 2018 | 15 | 50 | PC800 PROFESSIONAL |
| 22 | 2018 | 5 | 75 | PC800 PROFESSIONAL |
| 29 | 2018 | 15 | 50 | PC800 PROFESSIONAL |
| 29 | 2018 | 5 | 75 | PC800 PROFESSIONAL |
| 30 | 2018 | 5 | unknown | PC85 RAPIDFIRE PRO |
| 33 | 2018 | 5 | unknown | PC85 RAPIDFIRE PRO |
| 47 | 2018 | 5 | unknown | PC85 RAPIDFIRE PRO |
| 53 | 2018 | 15 | 75 | PC85 RAPIDFIRE PRO |
| 53 | 2018 | 5 | 75 | PC85 RAPIDFIRE PRO |
| 57 | 2018 | 5 | unknown | PC85 RAPIDFIRE PRO |
| 75 | 2018 | 5 | unknown | PC85 RAPIDFIRE PRO |
| 77 | 2018 | 5 | unknown | PC85 RAPIDFIRE PRO |
| 78 | 2018 | 5 | unknown | PC85 RAPIDFIRE PRO |
| 80 | 2018 | 5 | unknown | PC85 RAPIDFIRE PRO |
| 97 | 2018 | 15 | unknown | UltraFire |
| 147 | 2018 | 15 | 100 | HC600 HYPERFIRE |
| 4 | 2019 | 1 | 75 | PC800 PROFESSIONAL |
| 8 | 2019 | 1 | 100 | PC85 RAPIDFIRE PRO |
| 12 | 2019 | 1 | 75 | PC800 PROFESSIONAL |
| 23 | 2019 | 3 | 75 | PC800 PROFESSIONAL |
| 28 | 2019 | 5 | 75 | HC600 HYPERFIRE |
| 30 | 2019 | 3 | 75 | PC800 PROFESSIONAL |
| 37 | 2019 | 1 | 75 | PC85 RAPIDFIRE PRO |
| 39 | 2019 | 1 | 75 | PC85 RAPIDFIRE PRO |
| 40 | 2019 | 1 | 75 | PC800 PROFESSIONAL |
| 45 | 2019 | 1 | 75 | PC800 PROFESSIONAL |
| 52 | 2019 | 3 | 75 | PC800 PROFESSIONAL |
| 58 | 2019 | 1 | 75 | PC85 RAPIDFIRE PRO |
| 59 | 2019 | 15 | 75 | HC600 HYPERFIRE |
| 61 | 2019 | 1 | 75 | PC85 RAPIDFIRE PRO |
| 75 | 2019 | 1 | unknown | PC85 RAPIDFIRE PRO |
| 80 | 2019 | 3 | 75 | PC85 RAPIDFIRE PRO |
| 105 | 2019 | 1 | 75 | PC85 RAPIDFIRE PRO |
| 151 | 2019 | 1 | 75 | PC800 PROFESSIONAL |
